# National Mortality Trends, Disparities, and Forecasted Burden of Atrial Fibrillation and Heart Failure with Underlying Ischemic Heart Disease Among Older Adults in the United States, 1999–2023

**DOI:** 10.1007/s44197-026-00535-w

**Published:** 2026-03-19

**Authors:** Faizan Ahmed, Muhammad Abdullah, Haris Bin Tahir, Haider Hussain Shah, Maheen Sheraz, Muhammad Faizan Tahir, Umer Sajid, Tehmasp Rehman Mirza, Kinza Raza, Mohamed Bakr, Swapnil Patel, Fawaz Alenezi

**Affiliations:** 1 Jersey Shore University Medical Center, Neptune, NJ USA; 2 Shalamar Medical and Dental College, Lahore, Pakistan; 3 Lahore General Hospital, Lahore, Pakistan; 4 Bayhealth Hospital, Kent Campus, Dover, DE USA; 5 Continental Medical College, Lahore, Pakistan; 6 Rashid Latif Medical College, Lahore, Pakistan; 7 Bakhtawar Amin Medical & Dental College, Multan, Pakistan; 8 Duke University School of Medicine, Durham, NC USA

**Keywords:** Atrial fibrillation, Congestive heart failure, Ischemic heart disease, Age-adjusted mortality rates, Cardiovascular health disparities

## Abstract

**Background:**

Atrial fibrillation (AF) and congestive heart failure (CHF) remain major contributors to cardiovascular mortality in the United States. When accompanied by underlying ischemic heart disease (IHD), mortality risk is amplified. Although IHD mortality declined in the early 2000s, recent data suggest a resurgence.

**Methods:**

This population-based study used the CDC Wide-Ranging Online Data for Epidemiologic Research (CDC WONDER) database from 1999 to 2023. Deaths were identified using ICD-10 codes I48 (AF) and I50.0 (CHF) as multiple causes of death and I20–I25 (IHD) as the underlying cause. Adults aged ≥ 65 years were included. Crude mortality rates (CMRs) and age-adjusted mortality rates (AAMRs) were calculated. Temporal trends were analyzed using Joinpoint regression to estimate annual percent change (APC) and average annual percent change (AAPC). Future mortality trends were projected using autoregressive integrated moving average (ARIMA) model.

**Results:**

From 1999 to 2023, AF, CHF, and IHD accounted for 219,188 deaths among adults ≥ 65 years. The overall AAMR increased from 193.8 per million in 1999 to 220.1 per million in 2023, with a decline from 1999 to 2010 followed by a sustained rise. Males (108,732 deaths) showed the steepest increase (AAPC + 1.40%, *p* < 0.001), whereas females (110,456 deaths) experienced a modest decline (AAPC − 0.55%, *p* < 0.001). Non-Hispanic Black adults had the largest racial increase (AAPC + 1.21%, *p* < 0.001). Non-metropolitan areas showed the greatest geographic rise (AAPC + 1.67%, *p* < 0.001).

**Conclusion:**

Mortality involving AF and CHF with underlying IHD among U.S. adults ≥ 65 years declined only temporarily before reversing after 2010. Persistent racial and geographic disparities underscore gaps in prevention and acute cardiovascular care.

**Clinical Trial Registration:**

Not applicable.

**Supplementary Information:**

The online version contains supplementary material available at 10.1007/s44197-026-00535-w.

## Introduction

Ischemic heart disease (IHD) remains a leading cause of mortality in the United States despite decades of advances in prevention and revascularization strategies [1]. Survivors of ischemic injury frequently develop structural and electrical remodeling that predisposes them to chronic complications, including congestive heart failure (CHF) and atrial fibrillation (AF) [2, 3]. The coexistence of AF and CHF reflects a complex bidirectional relationship in which hemodynamic impairment, neurohormonal activation, and atrial remodeling accelerate disease progression and worsen prognosis [3]. In the setting of underlying ischemic substrate, this combination is associated with substantially elevated risks of hospitalization and death.

National mortality analyses have reported rising counts and age-adjusted rates of deaths in which AF is listed as a contributing cause, with ischemic heart disease frequently appearing as the underlying etiology [4]. Similarly, heart failure–related mortality among individuals with AF has increased over the past two decades [5]. More recent CDC WONDER–based investigations further demonstrate widening demographic and geographic disparities. Rani et al. [6] documented significant increases in AF/AFL-related heart failure mortality with marked variation by sex, race/ethnicity, region, and urbanization. Khan et al. [7] likewise reported steadily increasing hypertension-associated AF mortality across demographic groups. Together, these findings underscore the expanding public health burden of AF-related cardiovascular mortality.

Although prior studies have evaluated AF- or heart failure–related deaths, contemporary analyses specifically examining mortality in which ischemic heart disease serves as the underlying cause alongside coexisting AF and CHF remain limited. This distinction is clinically important, as IHD provides the structural substrate that frequently drives both arrhythmic and pump-failure complications. Moreover, older adults bear the highest absolute mortality burden from cardiovascular disease, and recent data suggest concerning reversals in long-standing declines in heart failure–related mortality within this population [8].

Accordingly, we conducted a comprehensive national analysis using CDC WONDER data from 1999 to 2023 to: [1] quantify long-term mortality trends involving atrial fibrillation and congestive heart failure with underlying ischemic heart disease among U.S. adults aged ≥ 65 years; [2] examine sex, racial/ethnic, and urban–rural disparities; and [3] project future mortality burden using time-series modeling. By focusing on this high-risk older population and etiologic framework, this study aims to clarify evolving national patterns and inform targeted cardiovascular prevention strategies.

## Methods

### Data Source and Study Design

We conducted a retrospective population-based mortality analysis using the National Center for Health Statistics (NCHS) Multiple Cause of Death Public Use Files accessed through the Centers for Disease Control and Prevention Wide-Ranging Online Data for Epidemiologic Research (CDC WONDER) database [9]. Death certificate data from all 50 U.S. states and the District of Columbia were examined for the period January 1, 1999 through December 31, 2023.

Deaths were eligible for inclusion if ischemic heart disease (ICD-10 codes I20–I25) was listed as the underlying cause of death and both atrial fibrillation/flutter (ICD-10 code I48) and congestive heart failure (ICD-10 code I50) were recorded as multiple (contributing) causes on the same death certificate [9]. Analyses were restricted to decedents aged 65 years and older at the time of death.

Because this study used publicly available, de-identified data, institutional review board approval was not required. The study adhered to the Strengthening the Reporting of Observational Studies in Epidemiology (STROBE) reporting guidelines [10].

### Variables and Stratification

Mortality data were stratified by sex, age group (65–74, 75–84, and ≥ 85 years), race/ethnicity, U.S. Census region, state of residence, urban–rural classification, and place of death. Race and ethnicity were categorized as non-Hispanic White, non-Hispanic Black or African American, non-Hispanic Asian or Pacific Islander, non-Hispanic American Indian or Alaska Native, and Hispanic or Latino, consistent with NCHS classification standards [11]. Geographic regions were defined according to U.S. Census Bureau classifications as Northeast, Midwest, South, and West [11].

Urban–rural status was determined using the 2013 National Center for Health Statistics Urban–Rural Classification Scheme for Counties [11]. Counties were categorized as large metropolitan (≥ 1,000,000 population), medium/small metropolitan (50,000–999,999 population), and non-metropolitan (< 50,000 population) based on the 2013 U.S. Census framework [11]. Place of death was classified as medical facility (inpatient, outpatient/emergency department, or death on arrival), nursing home/long-term care facility, hospice, decedent’s home, or other/unknown location.

### Mortality Rate Calculation

Annual crude mortality rates (CMRs) and age-adjusted mortality rates (AAMRs) per 1,000,000 population were calculated for the study period. Age adjustment was performed using the direct standardization method to the 2000 U.S. standard population to account for temporal changes in age distribution [12]. Suppressed counts (< 10 deaths), as reported by CDC WONDER, were handled in accordance with database reporting policies [9].

### Trend Analysis

Temporal trends in age-adjusted mortality rates from 1999 to 2023 were evaluated using the Joinpoint Regression Program (version 4.9.0.0, National Cancer Institute) [10]. Log-linear regression models were fitted to identify statistically significant changes in mortality trends over time. Annual percent change (APC) was calculated for each identified segment, and average annual percent change (AAPC) was computed to summarize overall trends across the study period. Statistical significance was determined using two-sided tests, with a p-value < 0.05 considered significant [10].

### Forecast Modeling

To estimate future mortality burden, autoregressive integrated moving average (ARIMA) models were applied to historical age-adjusted mortality rate data to generate projections through 2035. Model parameters were selected based on assessment of autocorrelation structure and minimization of the Akaike information criterion. Model diagnostics were evaluated to assess adequacy of fit. Forecasted age-adjusted mortality rates and corresponding 95% confidence intervals were calculated for overall and sex-stratified trends.

## Results

### Overall

**Fig. 1:**
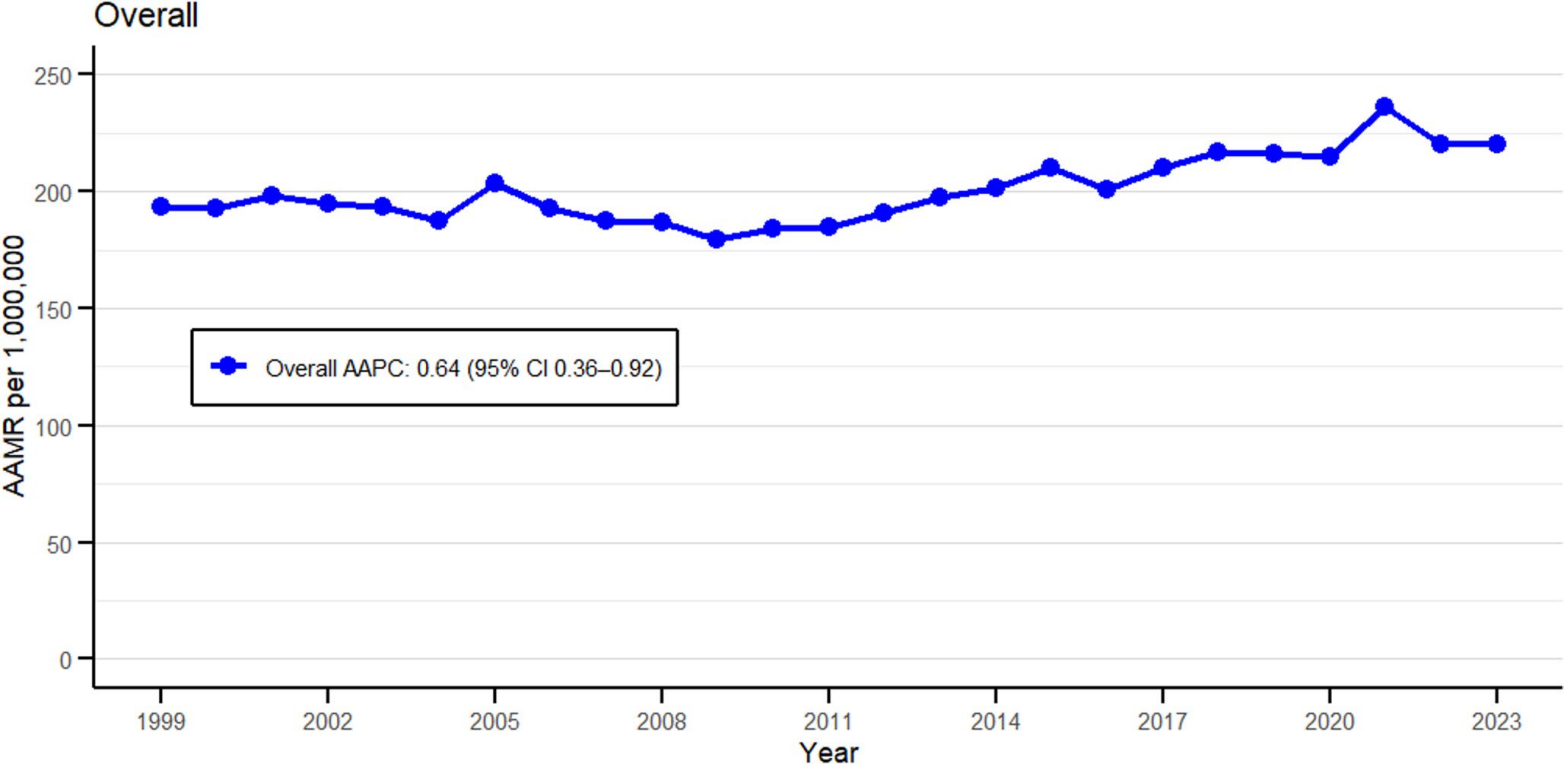
Overall age-adjusted mortality rate (AAMR) per 1,000,000 for atrial fibrillation, congestive heart failure, and ischemic heart disease in the United States, 1999–2023

Atrial Fibrillation/Atrial Flutter (AF/AFL) and Congestive heart failure (CHF) under ischemic heart disease (IHD) together caused a total of 219,188 deaths in the United States from 1999 to 2023 (Supplementary Table 1). The age-adjusted mortality rates (AAMR) per million ranged from 193.78 (95% CI 189.12–198.45) in 1999 to 220.11 (95% CI 216.08–224.13) in 2023, reaching its peak (Fig. 1). The average AAMR over the period was 200.65. Mortality rates revealed a marked increase over the study period, with an average annual percentage change (AAPC) of 0.64% (95% CI 0.36–0.91; *p* < 0.001) (Supplementary Table 2). The major upward spike was observed from 2010 to 2023, with an APC of 1.60% (95% CI 1.11–2.55; *p* < 0.001) (Supplementary Table 3).

### Sex

**Fig. 2:**
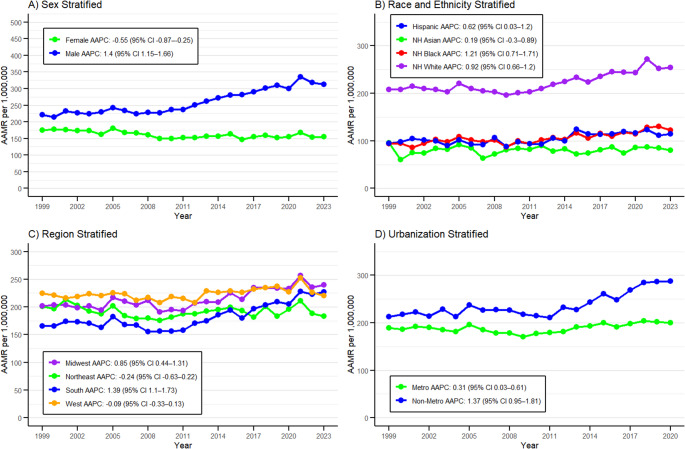
Age-adjusted mortality rates (AAMRs) per 1,000,000 population for atrial fibrillation, congestive heart failure, and ischemic heart disease in the United States, 1999–2023, stratified by sex (**A**), race and ethnicity (**B**), region (**C**), and urbanization status (**D**)

Stratification by sex revealed consistently higher AAMRs among males compared to females. From 1999 to 2023, females accounted for 110,456 deaths with an average AAMR of 161.71 reaching the highest level in 2005 at 180.37 (95% CI 175.02–185.71). Males accounted for fewer deaths at 108,732 with an average AAMR of 259.79 and peaking in 2021 at 335.22 (95% CI 327.09–343.35). Female population showed a decreased mortality trend from 1999 to 2023, while males demonstrated the opposite (Fig. 2A, Supplementary Table 2). The overall trend for females decreased with an AAPC of − 0.55% (95% CI − 0.87 to − 0.25; *p* < 0.001), while males showed an increase with an AAPC of 1.40% (95% CI 1.15–1.66; *p* < 0.001). Female population observed their greatest decline from 1999 to 2011 with an APC of − 1.37% (95% CI − 3.58 to − 0.73; *p* < 0.001), whereas the male population showed their greatest incline from 2009 to 2021 with an APC of 2.89% (95% CI 2.54–4.45; *p* < 0.001) (Supplementary Table 3).

### Race/Ethnicity

Database considering mortality trends was segregated into two main categories: Hispanic or Latino and Non-Hispanic Origin, which included four races (American Indian or Alaska Native, Asian or Pacific Islander, Black or African American, White). Among non-Hispanic groups, the highest average AAMR was observed among NH White at 222.04 peaking in 2021 at 271.54 (95% CI 266.26–276.81). This was followed by Black or African American individuals who had an average AAMR of 105.34 with a peak in 2022 at 130.23 (95% CI 119.69–140.77). Asian or Pacific Islander individuals had an average AAMR of 80.33 with a peak of 95.64 (95% CI 72.05–124.49) in 1999. American Indian or Alaska Native has suppressed AAMR values. Hispanic or Latino populations had an average AAMR of 104.58 with the highest value in 2015 at 124.76 (95% CI 112.78–136.75) (Fig. 2B, Supplementary Table 4). The highest AAPC was observed in Black or African American (1.20%; 95% CI 0.70–1.71; *p* < 0.001) with the greatest rise (APC 2.12%; 95% CI 1.34–6.01) seen between 2011 and 2023. This was followed by NH White with an AAPC of 0.92% (95% CI 0.65–1.19; *p* < 0.001) with the greatest rise (APC 2.02%; 95% CI 1.54–2.82) seen between 2010 and 2023. For Hispanic or Latino populations, the AAPC was 0.62% (95% CI 0.03–1.20; *p* < 0.001) with the greatest APC of 7.68% (95% CI 1.90–11.20) between 2012 and 2015 (Supplementary Table 3).

### Geographical Regions (Census & States)

Database considering mortality trends was segregated into four geographical areas i.e. Northeast, Midwest, South and Western regions. The highest number of casualties were reported in the South (71,241) followed by Midwest (53,717), West (51,500) and Northeast (42,730) from 1999 to 2023. The highest average AAMR per million was recorded in the West at 223.73 while the South region had the lowest, recorded at 182.10 between 1999 and 2023 (Fig. 2C, Supplementary Table 5). The Midwest (AAPC 0.85%; 95% CI 0.44 − 1.30; *p* < 0.001) and South (AAPC 1.39%; 95% CI 1.09–1.73; *p* < 0.001) had a rise in AAPC between 1999 and 2023. Whereas the Northeast (AAPC − 0.24%; 95% CI − 0.63 to 0.22; *p* < 0.001) and West (AAPC − 0.09%; 95% CI − 0.33 to 0.13; *p* < 0.001) regions showed a decline in their AAPCs (Supplementary Table 3).

From 1999 to 2023, the highest number of deaths related to the conditions were reported in California with 27,242 deaths and AAMR at 224.92 while the lowest were in the District of Columbia with 218 deaths and AAMR at 113.57 (Supplementary Table 6). The states having mortality rates at or above the 90th percentile were Rhode Island (377.17), Vermont (350.62), South Dakota (331.13), Washington (327.16), Oklahoma (310.50) and Idaho (307.56). The states having mortality rates at or below the 10th percentile were Connecticut (144.50), Alabama (138.38), Hawaii (135.84), Nevada (128.79), Georgia (121.58) and District of Columbia (113.57) (Supplementary Table 7).

### Urbanization

Segregation of the database by urbanization revealed that in the years 1999–2020, urban areas had the larger number of deaths (144,517) with the average AAMR of 188.71 with the highest AAMR reported in 2018 at 203.52 (95% CI 199.18–207.86) (Supplementary Table 6). Compared to this, rural areas had way less deaths (39,756) but a higher average AAMR of 236.80 with the highest value reported in 2020 at 287.62 (95% CI 276.35–298.89) (Fig. 2D, Supplementary Table 8). Both urban and rural areas had an overall increase in mortality trends from 1999 to 2020 with AAPCs of 0.30% (95% CI 0.03–0.61; *p* < 0.001) and 1.37% (95% CI 0.94–1.80; *p* < 0.001), respectively (Supplementary Table 3).

### Place of Death

Mortality trends based on place of death revealed that from 1999 to 2023, Nursing Home/Long term care had the greatest number of deaths (69,299), with the highest number of deaths in 2019 (3,192) and the lowest in 2004 (2,444). It was followed by Decedent’s home (63,629 deaths with the highest in 2021 with 4,461 and lowest in 2000 with 1,206), Medical Facility – Inpatient (53,329 deaths, highest in 2005 with 2,423 and lowest in 2020 with 1,863), Medical Facility – Outpatient or ER (12,237 deaths, highest in 2019 with 675 and lowest in 2001 with 355), Other (9,554 deaths, highest in 2022 with 668 and lowest in 1999 with 162), and Medical Facility – Dead on Arrival (833 deaths, highest in 2000 with 68 and lowest in 2017 with 14). Categories like Medical Facility – Status Unknown, Hospice Facility and Place of death unknown reported suppressed or zero deaths across the years (Supplementary Table 9).

### Forecast

**Fig. 3:**
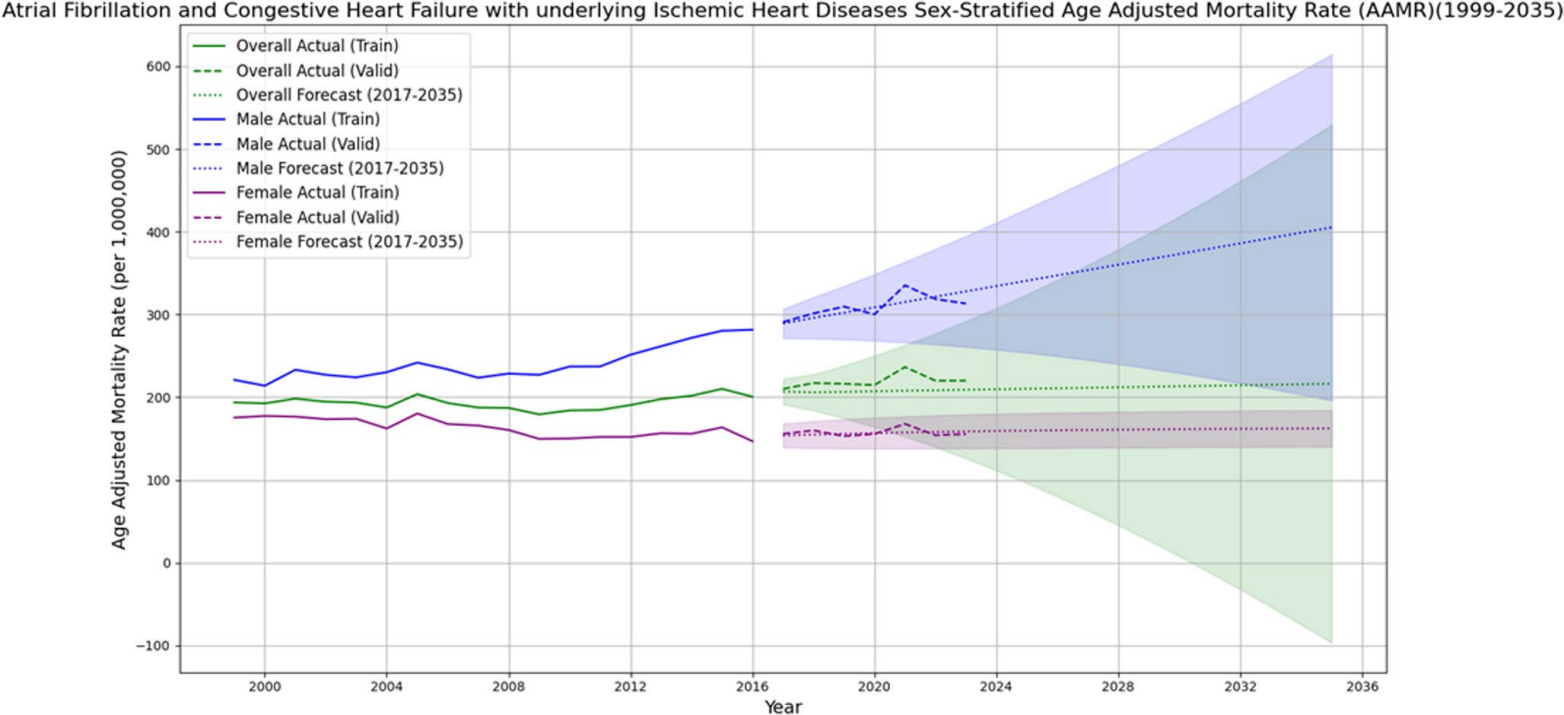
Age-adjusted mortality rates (AAMRs) per 1,000,000 population for atrial fibrillation, congestive heart failure, and ischemic heart disease in the United States from 1999 to 2023, with ARIMA model–based forecasts through 2035

**Fig. 4:**
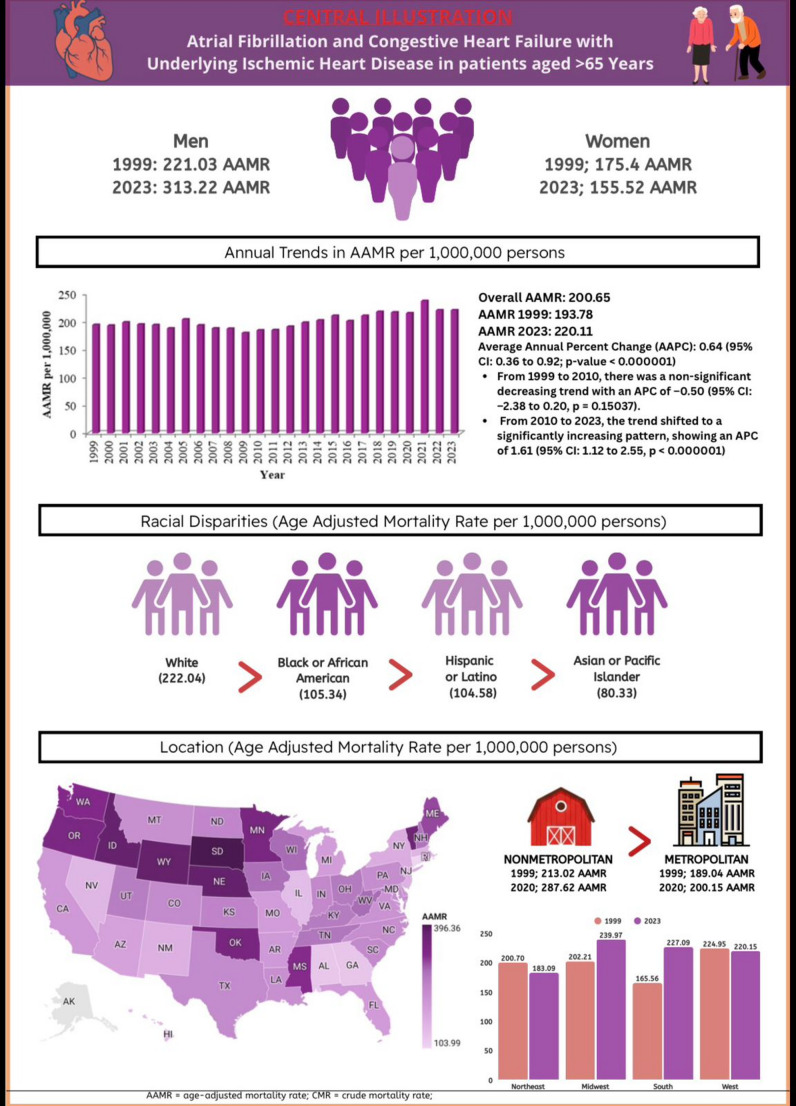
Central Illustration. Demographic profiles and temporal trends in mortality attributed to atrial fibrillation, congestive heart failure, and ischemic heart disease among adults aged ≥ 65 years in the United States, 1999–2023

Forecasting database trends till 2035 reveals rising mortality trends with Overall AAMR peaking at 216.31 in 2035 (95% CI − 96.67 to 529.29). Trends forecasted based on gender shows a similar trend and significant rise in AAMRs peaking in 2035 at 405.31 (95% CI 196.24–614.37) for males and 162.40 (95% CI 140.38–184.42) for females. Overall mortality trends increased significantly till 2035 with an AAPC of 0.28% (95% CI 0.275–0.285; *p* < 0.001). Mortality trends in males showed a steeper rise during the same period with an AAPC of 1.76% (95% CI 1.75–1.77; *p* < 0.001), while females demonstrated a slightly flatter upward trend with an AAPC of 0.18% (95% CI 0.17–0.18; *p* < 0.001) (Fig. 3). The central illustration highlights demographic patterns and long-term trends in mortality from atrial fibrillation, congestive heart failure, and ischemic heart disease among older adults in the United States between 1999 and 2023 (Fig. 4).

## Discussion

The present national analysis demonstrates a sustained rise in mortality involving atrial fibrillation/flutter and congestive heart failure when ischemic heart disease is the underlying cause among U.S. adults aged ≥ 65 years between 1999 and 2023. Although major advances in cardiovascular therapeutics have occurred during this period, age-adjusted mortality rates increased overall, particularly after 2010. These findings suggest that improvements in procedural and pharmacologic management have not fully offset the growing population-level burden of chronic cardiovascular disease.

Several factors likely contribute to this pattern. The aging of the U.S. population has substantially increased the number of individuals living with structural heart disease and its long-term sequelae, including atrial arrhythmias and heart failure [13]. Improved survival after myocardial ischemia may paradoxically expand the pool of patients at risk for subsequent electrical and mechanical complications [14]. The coexistence of AF and CHF reflects shared pathophysiologic pathways involving atrial remodeling, neurohormonal activation, and hemodynamic compromise, which collectively amplify morbidity and mortality risk [15, 16]. In parallel, the increasing prevalence of cardiometabolic risk factors—particularly obesity, diabetes, and hypertension—continues to drive both ischemic heart disease and arrhythmic burden [17]. At a population level, enhanced recognition and documentation of AF on death certificates may also partially contribute to observed increases in mortality coding [18].

A persistent sex disparity was observed, with males exhibiting consistently higher age-adjusted mortality rates than females. This pattern is consistent with broader epidemiologic evidence indicating a higher burden of ischemic heart disease and earlier onset of cardiovascular risk factors among men [19]. Biological differences, including the protective vascular and electrophysiologic effects of estrogen prior to menopause, may contribute to delayed disease manifestation in women [20]. Men are more likely to develop hypertension, diabetes, and dyslipidemia at younger ages and to engage in high-risk behaviors such as tobacco and excessive alcohol use, which accelerate atherosclerotic progression [21, 22]. Experimental and clinical studies have further demonstrated sex-based differences in inflammatory response and vascular remodeling during atherosclerosis [23, 24]. Although contemporary guidelines emphasize sex-specific risk recognition and preventive strategies [25, 26], disparities in risk factor control and health-seeking behavior may continue to influence mortality differences [27].

Racial and ethnic differences in mortality were also evident. Non-Hispanic White individuals demonstrated the highest age-adjusted mortality rates, consistent with prior epidemiologic observations of greater documented AF prevalence in this population [28, 29]. Differences in genetic susceptibility, atrial structure, and vascular risk factor expression may contribute to this distribution [30, 31]. However, longer life expectancy and greater access to diagnostic services may increase the likelihood of AF detection and cause-of-death attribution among White populations [32]. Conversely, underdiagnosis, disparities in cardiovascular care access, and differential insurance coverage patterns may contribute to lower recorded mortality rates in some minority populations [33]. These findings reinforce the importance of equitable preventive care and culturally responsive cardiovascular management strategies.

Geographic variation further underscores disparities in disease burden. The Western region demonstrated the highest age-adjusted mortality rates, with California contributing a substantial proportion of deaths. Demographic aging patterns and population growth in this region likely increase the absolute number of individuals at risk [34, 35]. Environmental exposures, including ambient air pollution, have been associated with both atrial fibrillation episodes and cardiovascular mortality [36]. Short-term exposure to particulate matter has been linked to increased AF incidence even at levels below current air quality thresholds [37], suggesting that environmental factors may play a contributory role. Additionally, regional differences in healthcare utilization and reporting practices may influence mortality attribution patterns [38].

Urban–rural disparities were also apparent. Although metropolitan areas accounted for a larger number of deaths, non-metropolitan regions demonstrated higher age-adjusted mortality rates. Rural populations frequently face barriers including limited specialty access, shortages of electrophysiology services, transportation constraints, and reduced availability of advanced cardiovascular therapies [39]. Insurance coverage differences, particularly in Medicaid and Medicare enrollment and outpatient cardiovascular care utilization, may further widen outcome gaps [40]. Comorbidity burden in older adults, including multimorbidity patterns observed in primary care populations, also complicates disease management and increases mortality risk [41]. In long-term care settings, high prevalence of frailty and comorbid illness, combined with emphasis on comfort-focused care, may contribute to higher observed mortality in nursing home residents [42].

Public health implications of these findings are substantial. Community-based cardiovascular prevention initiatives, particularly those targeting cardiometabolic risk reduction, may mitigate long-term arrhythmic and heart failure complications [43]. Continued investment in surveillance systems is essential to monitor evolving mortality trends and evaluate intervention effectiveness [44]. Environmental policy interventions aimed at reducing ambient air pollution may also yield cardiovascular health benefits, especially in densely populated regions [45].

### Limitations

This study has several limitations inherent to administrative mortality data. Cause-of-death classification relies on ICD-10 coding and may be subject to misclassification or variability in documentation practices. The temporal relationship between atrial fibrillation, heart failure, and ischemic heart disease cannot be determined from death certificate data alone. Individual-level clinical information, including severity of disease, treatment patterns, socioeconomic status, and genetic factors, was unavailable. Residual confounding due to unmeasured risk factors cannot be excluded. Finally, geographic variation in reporting practices and healthcare access may influence observed mortality differences.

## Conclusion

Mortality involving atrial fibrillation and congestive heart failure in the setting of underlying ischemic heart disease has increased among older adults in the United States over the past two decades, with marked disparities by sex, race/ethnicity, geography, and urbanization status. Despite therapeutic advancements, the persistent upward trend underscores the continuing impact of population aging, cardiometabolic risk burden, and inequities in access to care. Strengthened prevention strategies, equitable healthcare delivery, environmental risk mitigation, and continued national surveillance are critical to reducing future cardiovascular mortality in this high-risk population.

## Supplementary Information

Below is the link to the electronic supplementary material.

Supplementary Material 1 (DOCX 38.5 KB)

## Data Availability

All data used in this study are publicly available through the Centers for Disease Control and Prevention Wide-ranging Online Data for Epidemiologic Research (CDC WONDER) database. The authors obtained access in accordance with the CDC’s data use guidelines.

## Declarations

### Permission to Reproduce Material from Other Sources

Not applicable. No third-party material was reproduced.

### Artificial Intelligence (AI) Use Statement

The authors declare that no generative artificial intelligence (AI) tools were used in the preparation, writing, editing, or analysis of this manuscript. All content was prepared and reviewed by the authors based on their own clinical observations and relevant published studies. All references were individually verified through PubMed and CrossRef, confirming the authenticity and accuracy of each cited source. No fabricated, AI-generated, or unverifiable references were included in this work. 
