# Supplementary material for: National Mortality Trends, Disparities, and Forecasted Burden of Atrial Fibrillation and Heart Failure with Underlying Ischemic Heart Disease Among Older Adults in the United States, 1999–2023
Source: J Epidemiol Glob Health. 2026 Mar 19;16(1):47. doi: 10.1007/s44197-026-00535-w (PMC13057089; doi:10.1007/s44197-026-00535-w)
Supplement: Supplementary file 1 — Supplementary Material 1 (DOCX 38.5 KB) [file 44197_2026_535_MOESM1_ESM.docx]

**Supplementary Data**

**Supplementary Table 1. Number of Atrial Fibrillation and Flutter and Congestive Heart Failure under Ischemic Heart Disease-Related Deaths, Overall and stratified by Sex and Race in Adults in the United States 1999-2023.**

|  | **Deaths** | | | | | | |
| --- | --- | --- | --- | --- | --- | --- | --- |
| **Year** | **Overall** | **Women** | **Men** | **NH Asian or Pacific Islander** | **NH Black or African American** | **NH White** | **Hispanic or Latino** |
| **1999** | 6624 | 4001 | 2623 | 57 | 246 | 6163 | 124 |
| **2000** | 6683 | 4108 | 2575 | 38 | 251 | 6231 | 136 |
| **2001** | 6993 | 4128 | 2865 | 53 | 228 | 6538 | 154 |
| **2002** | 6946 | 4104 | 2842 | 56 | 254 | 6445 | 154 |
| **2003** | 7026 | 4164 | 2862 | 67 | 277 | 6492 | 164 |
| **2004** | 6902 | 3910 | 2992 | 71 | 264 | 6393 | 154 |
| **2005** | 7662 | 4430 | 3232 | 85 | 304 | 7067 | 184 |
| **2006** | 7428 | 4204 | 3224 | 84 | 287 | 6855 | 179 |
| **2007** | 7396 | 4239 | 3157 | 67 | 286 | 6837 | 185 |
| **2008** | 7527 | 4180 | 3347 | 80 | 303 | 6894 | 229 |
| **2009** | 7379 | 3972 | 3407 | 95 | 268 | 6785 | 204 |
| **2010** | 7687 | 4046 | 3641 | 104 | 313 | 7018 | 232 |
| **2011** | 7966 | 4182 | 3784 | 113 | 305 | 7270 | 244 |
| **2012** | 8421 | 4274 | 4147 | 135 | 348 | 7635 | 258 |
| **2013** | 8976 | 4501 | 4475 | 127 | 378 | 8125 | 311 |
| **2014** | 9329 | 4542 | 4787 | 144 | 379 | 8435 | 316 |
| **2015** | 9923 | 4812 | 5111 | 138 | 445 | 8876 | 421 |
| **2016** | 9662 | 4401 | 5261 | 147 | 417 | 8634 | 411 |
| **2017** | 10358 | 4751 | 5607 | 174 | 478 | 9225 | 429 |
| **2018** | 10976 | 4980 | 5996 | 194 | 471 | 9810 | 457 |
| **2019** | 11145 | 4811 | 6334 | 179 | 518 | 9897 | 498 |
| **2020** | 11264 | 4946 | 6318 | 219 | 531 | 9943 | 507 |
| **2021** | 11680 | 4965 | 6715 | 210 | 570 | 10263 | 519 |
| **2022** | 11660 | 4953 | 6707 | 226 | 608 | 10218 | 506 |
| **2023** | 11575 | 4852 | 6723 | 216 | 573 | 10134 | 538 |
| **Total** | 219188 | 110456 | 108732 | 3079 | 9302 | 198183 | 7514 |

**Supplementary Table 2. Atrial Fibrillation and Flutter and Congestive Heart Failure under Ischemic Heart Disease related Age-Adjusted Mortality Rates per 1,000,000 Overall and Stratified by Sex in Adults in the United States 1999-2023**

| **Age-Adjusted Rate (95% CI)** | | | |
| --- | --- | --- | --- |
| Year | **Men** | **Women** | **Overall** |
| **1999** | 221.03 (212.42-229.64) | 175.4 (169.95-180.85) | 193.78 (189.12-198.45) |
| **2000** | 213.96 (205.56-222.36) | 177.46 (172.01-182.9) | 192.58 (187.97-197.2) |
| **2001** | 233.15 (224.48-241.82) | 176.61 (171.2-182.02) | 198.29 (193.64-202.93) |
| **2002** | 227.11 (218.64-235.58) | 173.49 (168.16-178.82) | 194.65 (190.07-199.23) |
| **2003** | 224.07 (215.76-232.38) | 174.04 (168.73-179.35) | 193.6 (189.07-198.12) |
| **2004** | 230.3 (221.95-238.64) | 162.26 (157.15-167.37) | 187.61 (183.18-192.03) |
| **2005** | 241.88 (233.46-250.29) | 180.37 (175.02-185.71) | 203.78 (199.22-208.35) |
| **2006** | 233.75 (225.63-241.87) | 167.66 (162.56-172.77) | 193.05 (188.65-197.44) |
| **2007** | 223.62 (215.78-231.45) | 165.8 (160.77-170.84) | 187.59 (183.31-191.87) |
| **2008** | 228.63 (220.85-236.41) | 160.47 (155.55-165.38) | 187.13 (182.89-191.37) |
| **2009** | 227.07 (219.42-234.72) | 149.72 (145.01-154.43) | 179.38 (175.27-183.48) |
| **2010** | 237.13 (229.41-244.86) | 150.2 (145.51-154.89) | 184.13 (179.99-188.26) |
| **2011** | 237.19 (229.61-244.77) | 152.01 (147.33-156.69) | 184.66 (180.58-188.74) |
| **2012** | 251.44 (243.76-259.12) | 152.01 (147.38-156.65) | 190.61 (186.51-194.7) |
| **2013** | 261.65 (253.95-269.35) | 156.56 (151.9-161.21) | 197.82 (193.7-201.95) |
| **2014** | 271.82 (264.08-279.56) | 155.98 (151.37-160.6) | 201.85 (197.73-205.98) |
| **2015** | 280.33 (272.6-288.07) | 163.67 (158.96-168.38) | 210.19 (206.02-214.36) |
| **2016** | 281.6 (273.95-289.26) | 146.93 (142.52-151.34) | 200.6 (196.57-204.64) |
| **2017** | 290.82 (283.16-298.49) | 155.81 (151.32-160.31) | 210.09 (206.02-214.17) |
| **2018** | 301.41 (293.73-309.09) | 159.94 (155.44-164.44) | 217.2 (213.11-221.28) |
| **2019** | 309.47 (301.8-317.14) | 153.07 (148.7-157.44) | 216.27 (212.24-220.31) |
| **2020** | 300.17 (292.71-307.63) | 155.63 (151.26-160.01) | 214.8 (210.81-218.78) |
| **2021** | 335.22 (327.09-343.35) | 167.86 (163.17-172.55) | 236.54 (232.23-240.85) |
| **2022** | 318.61 (310.89-326.32) | 154.17 (149.85-158.49) | 219.99 (215.98-223.99) |
| **2023** | 313.22 (305.63-320.82) | 155.52 (151.13-159.9) | 220.11 (216.08-224.13) |

**Supplementary Table 3 Annual Percent Change (APC) and Average Annual Percent Change (AAPC) of Atrial Fibrillation and Flutter and Congestive Heart Failure under Ischemic Heart Disease -related Age-Adjusted Mortality Rates per 1,000,000 in Adults in the United States 1999-2023**

| **Year Interval** | **APC (95% CI)** | **Year Interval** | **AAPC (95% CI)** |
| --- | --- | --- | --- |
| **Overall** |  |  |  |
| 1999-2010 | -0.4985 (-2.3752-0.2038) | 1999-2023 | 0.6365* (0.3637-0.9166) |
| 2010-2023 | 1.6069* (1.1172-2.547) |  |  |
| **Men** |  |  |  |
| 1999-2009 | 0.3139 (-0.8047-1.054) | 1999-2023 | 1.3987* (1.1471-1.6593) |
| 2009-2021 | 2.8994* (2.5488-4.4532) |  |  |
| 2021-2023 | -2.0268 (-4.7493-1.6269) |  |  |
| **Women** |  |  |  |
| 1999-2011 | -1.3679* (-3.5777--0.7375) | 1999-2023 | -0.5531* (-0.869--0.2476) |
| 2011-2023 | 0.2685 (-0.2983-2.3244) |  |  |
| **NH Asian or Pacific Islander** |  |  |  |
| 1999-2023 | 0.1912 (-0.302-0.8905) | 1999-2023 | 0.1912 (-0.302-0.8905) |
| **NH Black or African American** |  |  |  |
| 1999-2011 | 0.2906 (-5.0132-1.3162) | 1999-2023 | 1.2050* (0.7091-1.7126) |
| 2011-2023 | 2.1276* (1.3476-6.0169) |  |  |
| **NH White** |  |  |  |
| 1999-2010 | -0.3587 (-1.8414-0.3244) | 1999-2023 | 0.9228* (0.6554-1.1953) |
| 2010-2023 | 2.0200* (1.5428-2.8255) |  |  |
| **Hispanic or Latino** |  |  |  |
| 1999-2012 | -0.4417 (-4.8574-0.9073) | 1999-2023 | 0.6217* (0.0317-1.1986) |
| 2012-2015 | 7.6883* (1.9036-11.2049) |  |  |
| 2015-2023 | -0.1989 (-3.2431-1.2429) |  |  |
| **Census Region 1 – Northeast** |  |  |  |
| 1999-2007 | -1.5107* (-6.2963--0.128) | 1999-2023 | -0.2356 (-0.6315-0.219) |
| 2007-2023 | 0.4081 (-0.1029-2.9752) |  |  |
| **Census Region 2 – Midwest** |  |  |  |
| 1999-2011 | -0.0748 (-4.103-0.9184) | 1999-2023 | 0.8470* (0.4405-1.3058) |
| 2011-2023 | 1.7772* (0.9464-5.4136) |  |  |
| **Census Region 3 – South** |  |  |  |
| 1999-2005 | 0.8113 (-0.9053-5.3013) | 1999-2023 | 1.3909* (1.0957-1.728) |
| 2005-2009 | -2.9214 (-5.754-4.4639) |  |  |
| 2009-2023 | 2.9104* (1.701-3.7631) |  |  |
| **Census Region 4 – West** |  |  |  |
| 1999-2011 | -0.3018 (-1.3354-0.1495) | 1999-2023 | -0.0886 (-0.3306-0.1327) |
| 2011-2021 | 1.2069* (0.7667-3.0409) |  |  |
| 2021-2023 | -5.1139* (-7.8726--1.3196) |  |  |
| **Urban** |  |  |  |
| 1999-2009 | -0.8057* (-2.7456--0.0852) | 1999-2020 | 0.3054* (0.0275-0.6116) |
| 2009-2020 | 1.3263* (0.815-2.5786) |  |  |
| **Rural** |  |  |  |
| 1999-2011 | 0.0029 (-2.4202-0.8553) | 1999-2020 | 1.3727* (0.9461-1.8052) |
| 2011-2020 | 3.2283* (2.0978-6.7512) |  |  |

*=indicates statistically significant value (p < 0.05)

**Supplementary Table 4 Atrial Fibrillation and Flutter and Congestive Heart Failure under Ischemic Heart Disease -related Age-Adjusted Mortality Rates per 1,000,000, Stratified by Race in Adults in the United States 1999-2023**

|  | **Age-Adjusted Rate (95% CI)** | | | |
| --- | --- | --- | --- | --- |
| **Year** | **NH Asian or Pacific Islander** | **NH Black or African American** | **NH White** | **Hispanic or Latino** |
| **1999** | 95.64 (72.05-124.49) | 94.15 (82.35-105.95) | 208.15 (202.95-213.34) | 94.5 (77.62-111.38) |
| **2000** | 60.61 (42.67-83.54) | 95.17 (83.37-106.97) | 207.85 (202.69-213.01) | 97.48 (80.89-114.07) |
| **2001** | 74.86 (55.74-98.42) | 86.37 (75.13-97.61) | 215.27 (210.05-220.49) | 104.77 (88-121.54) |
| **2002** | 74.14 (55.69-96.73) | 95.26 (83.5-107.01) | 210.03 (204.9-215.16) | 101.63 (85.38-117.88) |
| **2003** | 83.79 (64.67-106.8) | 103.12 (90.93-115.31) | 208.47 (203.4-213.55) | 99.58 (84.13-115.03) |
| **2004** | 81.72 (63.58-103.42) | 97.53 (85.73-109.34) | 203.29 (198.3-208.28) | 89.72 (75.34-104.1) |
| **2005** | 92.18 (73.42-114.27) | 108.88 (96.6-121.16) | 220.58 (215.43-225.73) | 101.9 (87-116.8) |
| **2006** | 85.46 (68.07-105.94) | 101.65 (89.86-113.45) | 209.81 (204.83-214.78) | 93.08 (79.3-106.85) |
| **2007** | 63.61 (49.2-80.93) | 98.04 (86.64-109.44) | 204.91 (200.03-209.78) | 92.31 (78.9-105.72) |
| **2008** | 71.92 (56.94-89.63) | 101.79 (90.29-113.29) | 203.51 (198.68-208.33) | 106.74 (92.81-120.67) |
| **2009** | 81.4 (65.78-99.61) | 86.56 (76.15-96.96) | 196.63 (191.93-201.33) | 88.22 (76.02-100.42) |
| **2010** | 83.83 (67.66-100.01) | 99.39 (88.33-110.44) | 201.3 (196.56-206.04) | 97.99 (85.32-110.66) |
| **2011** | 81.72 (66.59-96.86) | 93.69 (83.13-104.24) | 203.18 (198.48-207.89) | 94.35 (82.47-106.24) |
| **2012** | 89.84 (74.6-105.07) | 101.52 (90.79-112.26) | 209.54 (204.79-214.28) | 92.84 (81.47-104.22) |
| **2013** | 77.94 (64.32-91.57) | 106.51 (95.7-117.32) | 218.75 (213.95-223.55) | 105.84 (94.03-117.64) |
| **2014** | 82.69 (69.12-96.25) | 102.97 (92.51-113.43) | 224.89 (220.04-229.74) | 99.71 (88.66-110.75) |
| **2015** | 72.67 (60.48-84.86) | 116.53 (105.59-127.46) | 233.5 (228.59-238.41) | 124.76 (112.78-136.75) |
| **2016** | 74.39 (62.31-86.48) | 106.16 (95.87-116.46) | 224.05 (219.28-228.82) | 114.82 (103.65-126) |
| **2017** | 81.51 (69.33-93.69) | 115.56 (105.08-126.04) | 235.73 (230.88-240.58) | 113.69 (102.86-124.52) |
| **2018** | 86.54 (74.28-98.79) | 109.36 (99.36-119.36) | 245.82 (240.93-250.72) | 114.82 (104.21-125.43) |
| **2019** | 74.21 (63.27-85.14) | 117.98 (107.7-128.26) | 244.81 (239.96-249.65) | 119.76 (109.17-130.35) |
| **2020** | 85.6 (74.19-97.01) | 114.56 (104.68-124.44) | 243.67 (238.86-248.48) | 116.68 (106.45-126.92) |
| **2021** | 87.38 (75.49-99.28) | 128.2 (117.46-138.94) | 271.54 (266.26-276.81) | 123.4 (112.71-134.09) |
| **2022** | 84.71 (73.6-95.81) | 130.23 (119.69-140.77) | 251.81 (246.92-256.71) | 111.18 (101.41-120.94) |
| **2023** | 79.88 (69.19-90.57) | 122.4 (112.18-132.61) | 253.99 (249.04-258.95) | 114.69 (104.9-124.49) |

**Supplementary Table 5 Atrial Fibrillation and Flutter and Congestive Heart Failure under Ischemic Heart Disease -related Age-Adjusted Mortality Rate per 1,000,000 Stratified by Census Region in Adults in the United States 1999-2023**

| **Age-Adjusted Rate (95% CI)** | | | | |
| --- | --- | --- | --- | --- |
| **Year** | **Northeast** | **Midwest** | **South** | **West** |
| **1999** | 200.7 (190.52-210.88) | 202.21 (192.61-211.82) | 165.56 (158.21-172.92) | 224.95 (213.48-236.42) |
| **2000** | 197.14 (187.13-207.14) | 203.59 (194-213.19) | 165.44 (158.14-172.74) | 221.03 (209.78-232.27) |
| **2001** | 213.72 (203.38-224.07) | 203.15 (193.63-212.67) | 174.3 (166.85-181.75) | 216.37 (205.41-227.33) |
| **2002** | 202.34 (192.34-212.34) | 198.26 (188.89-207.62) | 173.26 (165.86-180.66) | 218.24 (207.33-229.15) |
| **2003** | 192.29 (182.62-201.97) | 202.06 (192.67-211.44) | 170.38 (163.09-177.66) | 223.98 (213.07-234.9) |
| **2004** | 187.05 (177.55-196.56) | 194.45 (185.3-203.6) | 163.27 (156.2-170.35) | 220.18 (209.47-230.89) |
| **2005** | 201.73 (191.95-211.51) | 217.25 (207.64-226.87) | 182.01 (174.63-189.39) | 225.52 (214.85-236.19) |
| **2006** | 184.37 (175.09-193.66) | 210.11 (200.74-219.48) | 167.7 (160.7-174.69) | 223.82 (213.34-234.3) |
| **2007** | 178.78 (169.72-187.84) | 203.13 (194-212.27) | 167.37 (160.47-174.28) | 211.9 (201.86-221.95) |
| **2008** | 180.17 (171.14-189.2) | 212.06 (202.78-221.33) | 155.1 (148.54-161.67) | 217.35 (207.31-227.39) |
| **2009** | 175.93 (167.06-184.8) | 190.86 (182.1-199.62) | 156.02 (149.51-162.52) | 207.98 (198.3-217.67) |
| **2010** | 181.13 (172.17-190.1) | 195.09 (186.29-203.89) | 156.56 (150.1-163.03) | 218.63 (208.79-228.46) |
| **2011** | 187.18 (178.13-196.23) | 193.42 (184.74-202.1) | 158.17 (151.8-164.55) | 215.44 (205.85-225.03) |
| **2012** | 187.31 (178.32-196.29) | 206.97 (198.05-215.88) | 170.68 (164.15-177.21) | 207.62 (198.35-216.89) |
| **2013** | 192.28 (183.24-201.33) | 209.18 (200.31-218.05) | 174.45 (167.95-180.95) | 228.56 (218.96-238.15) |
| **2014** | 195.09 (186.01-204.17) | 208.72 (199.89-217.54) | 185.51 (178.89-192.13) | 226.3 (216.88-235.71) |
| **2015** | 199.33 (190.2-208.47) | 225.19 (216.1-234.29) | 194.43 (187.74-201.11) | 228.69 (219.35-238.03) |
| **2016** | 193.2 (184.22-202.17) | 213.65 (204.82-222.48) | 179.88 (173.55-186.22) | 226.34 (217.2-235.48) |
| **2017** | 181.4 (172.84-189.96) | 234.51 (225.35-243.66) | 196.63 (190.09-203.17) | 232.48 (223.31-241.66) |
| **2018** | 201.31 (192.42-210.2) | 234.44 (225.39-243.48) | 203.18 (196.65-209.72) | 234.36 (225.28-243.44) |
| **2019** | 183.47 (175.06-191.88) | 233.42 (224.44-242.4) | 209.41 (202.87-215.96) | 237.51 (228.5-246.52) |
| **2020** | 195.74 (187.05-204.43) | 233.31 (224.38-242.24) | 204.94 (198.55-211.32) | 227.51 (218.8-236.22) |
| **2021** | 210.73 (201.51-219.95) | 256.23 (246.57-265.9) | 228.32 (221.35-235.28) | 252 (242.52-261.47) |
| **2022** | 188.69 (180.22-197.16) | 235.17 (226.2-244.14) | 222.89 (216.28-229.5) | 226.32 (217.72-234.93) |
| **2023** | 183.09 (174.69-191.48) | 239.97 (230.83-249.11) | 227.09 (220.41-233.77) | 220.15 (211.64-228.66) |

**Supplementary Table 6 Atrial Fibrillation and Flutter and Congestive Heart Failure under Ischemic Heart Disease Related Deaths, Stratified by Census Region and Urbanization in Adults in the United States 1999-2023.**

|  | **Deaths** | | | | | |
| --- | --- | --- | --- | --- | --- | --- |
| **Years** | **Northeast** | **Midwest** | **South** | **West** | **Rural** | **Urban** |
| **1999** | *1494* | *1703* | *1948* | *1479* | *1434* | *5190* |
| **2000** | *1492* | *1732* | *1974* | *1485* | *1483* | *5200* |
| **2001** | *1641* | *1750* | *2105* | *1497* | *1522* | *5471* |
| **2002** | *1575* | *1724* | *2109* | *1538* | *1469* | *5477* |
| **2003** | *1520* | *1784* | *2104* | *1618* | *1576* | *5450* |
| **2004** | *1491* | *1738* | *2050* | *1623* | *1480* | *5422* |
| **2005** | *1641* | *1967* | *2338* | *1716* | *1664* | *5998* |
| **2006** | *1525* | *1941* | *2209* | *1753* | *1613* | *5815* |
| **2007** | *1510* | *1914* | *2258* | *1714* | *1647* | *5749* |
| **2008** | *1547* | *2026* | *2146* | *1808* | *1655* | *5872* |
| **2009** | *1534* | *1846* | *2216* | *1783* | *1615* | *5764* |
| **2010** | *1598* | *1913* | *2260* | *1916* | *1605* | *6082* |
| **2011** | *1684* | *1942* | *2375* | *1965* | *1609* | *6357* |
| **2012** | *1714* | *2112* | *2641* | *1954* | *1802* | *6619* |
| **2013** | *1789* | *2183* | *2790* | *2214* | *1808* | *7168* |
| **2014** | *1830* | *2197* | *3046* | *2256* | *1943* | *7386* |
| **2015** | *1888* | *2409* | *3284* | *2342* | *2119* | *7804* |
| **2016** | *1838* | *2300* | *3129* | *2395* | *2038* | *7624* |
| **2017** | *1777* | *2571* | *3508* | *2502* | *2250* | *8108* |
| **2018** | *2016* | *2621* | *3748* | *2591* | *2428* | *8548* |
| **2019** | *1862* | *2632* | *3955* | *2696* | *2479* | *8666* |
| **2020** | *1980* | *2653* | *3985* | *2646* | *2517* | *8747* |
| **2021** | *2029* | *2730* | *4176* | *2745* | **-** | **-** |
| **2022** | *1920* | *2662* | *4403* | *2675* | **-** | **-** |
| **2023** | *1835* | *2667* | *4484* | *2589* | **-** | **-** |
| **Total** | 42730 | 53717 | 71241 | *51500* | 39756 | 144517 |

**Supplementary Table 7 Atrial Fibrillation and Flutter and Congestive Heart Failure under Ischemic Heart Disease -related Age Adjusted Mortality Rates per 1,000,000 Stratified by States among Adults in the United States 1999-2023**

| **State** | **Rank** | **Deaths** | **Age Adjusted Rate** | **Percentile** |
| --- | --- | --- | --- | --- |
| Rhode Island | 1 | 1825 | 377.165 | 100 |
| Vermont | 2 | 820 | 350.615 | 98 |
| South Dakota | 3 | 990 | 331.125 | 96 |
| Washington | 4 | 7319 | 327.16 | 94 |
| Oklahoma | 5 | 3616 | 310.495 | 92 |
| Idaho | 6 | 1305 | 307.555 | 90 |
| Oregon | 7 | 4077 | 306.33 | 88 |
| Nebraska | 8 | 1722 | 299.98 | 86 |
| West Virginia | 9 | 2230 | 296.65 | 84 |
| Minnesota | 10 | 4915 | 289.21 | 82 |
| Ohio | 11 | 12928 | 285.56 | 80 |
| Wyoming | 12 | 387 | 284.21 | 78 |
| North Dakota | 13 | 894 | 277.62 | 76 |
| Iowa | 14 | 3506 | 271.38 | 73 |
| Maine | 15 | 1349 | 266.755 | 71 |
| Tennessee | 16 | 5339 | 262.98 | 69 |
| New Hampshire | 17 | 1211 | 262.25 | 67 |
| Mississippi | 18 | 2039 | 257.55 | 65 |
| Wisconsin | 19 | 4474 | 241.23 | 63 |
| Kentucky | 20 | 3151 | 240.3 | 61 |
| Pennsylvania | 21 | 12905 | 233.45 | 59 |
| Indiana | 22 | 4831 | 229.46 | 57 |
| Texas | 23 | 14523 | 229.085 | 55 |
| Delaware | 24 | 762 | 227.925 | 53 |
| California | 25 | 27242 | 224.915 | 51 |
| Colorado | 26 | 2938 | 220.22 | 49 |
| South Carolina | 27 | 3124 | 219.505 | 47 |
| North Carolina | 28 | 6434 | 218.6 | 45 |
| Utah | 29 | 1147 | 215.42 | 43 |
| Maryland | 30 | 3726 | 213.31 | 41 |
| Kansas | 31 | 1897 | 208.49 | 39 |
| Montana | 32 | 682 | 198.22 | 37 |
| Michigan | 33 | 6764 | 193.785 | 35 |
| Missouri | 34 | 3961 | 188.435 | 33 |
| Arkansas | 35 | 1815 | 187.915 | 31 |
| Virginia | 36 | 3886 | 183.52 | 29 |
| Louisiana | 37 | 1917 | 175.195 | 27 |
| Arizona | 38 | 3568 | 171.6 | 24 |
| Florida | 39 | 13164 | 170.035 | 22 |
| New York | 40 | 12324 | 169.62 | 20 |
| New Jersey | 41 | 5741 | 168.47 | 18 |
| New Mexico | 42 | 1127 | 166.98 | 16 |
| Massachusetts | 43 | 3913 | 152.735 | 14 |
| Illinois | 44 | 6835 | 150.275 | 12 |
| Connecticut | 45 | 2642 | 144.5 | 10 |
| Alabama | 46 | 2371 | 138.38 | 8 |
| Hawaii | 47 | 711 | 135.84 | 6 |
| Nevada | 48 | 807 | 128.79 | 4 |
| Georgia | 49 | 2926 | 121.58 | 2 |
| District of Columbia | 50 | 218 | 113.57 | 0 |

**Supplementary Table 8 Atrial Fibrillation and Flutter and Congestive Heart Failure under Ischemic Heart Disease -related Age-Adjusted Mortality Rates per 1,000,000 in the Rural and Urban areas in Adults in the United States 1999-2020**

|  | **Age-Adjusted Rate (95% CI)** | |
| --- | --- | --- |
| **Year** | **Urban** | **Rural** |
| **1999** | 189.04 (183.9-194.19) | 213.02 (201.99-224.05) |
| **2000** | 186.37 (181.3-191.44) | 217.9 (206.81-229) |
| **2001** | 192.42 (187.33-197.52) | 222.57 (211.39-233.76) |
| **2002** | 189.96 (184.93-194.99) | 214.26 (203.3-225.22) |
| **2003** | 185.47 (180.55-190.4) | 228.09 (216.83-239.35) |
| **2004** | 181.75 (176.91-186.59) | 213.1 (202.24-223.96) |
| **2005** | 196.14 (191.17-201.1) | 237.05 (225.66-248.45) |
| **2006** | 185.51 (180.74-190.28) | 226.22 (215.17-237.27) |
| **2007** | 178.6 (173.98-183.23) | 227.58 (216.58-238.59) |
| **2008** | 178.36 (173.79-182.93) | 226.47 (215.54-237.4) |
| **2009** | 170.89 (166.46-175.31) | 218.19 (207.52-228.85) |
| **2010** | 177.33 (172.86-181.81) | 214.81 (204.27-225.35) |
| **2011** | 179.11 (174.68-183.54) | 210.44 (200.12-220.76) |
| **2012** | 181.71 (177.3-186.12) | 232.03 (221.28-242.79) |
| **2013** | 191.56 (187.09-196.04) | 227.93 (217.38-238.48) |
| **2014** | 193.11 (188.67-197.55) | 243.62 (232.74-254.5) |
| **2015** | 199.77 (195.29-204.24) | 260.62 (249.47-271.77) |
| **2016** | 190.92 (186.59-195.24) | 247.86 (237.05-258.66) |
| **2017** | 197.98 (193.64-202.33) | 268.98 (257.82-280.14) |
| **2018** | 203.52 (199.18-207.86) | 284.45 (273.1-295.8) |
| **2019** | 201.94 (197.67-206.22) | 286.79 (275.47-298.12) |
| **2020** | 200.15 (195.93-204.36) | 287.62 (276.35-298.89) |

**Supplementary Table 9 Atrial Fibrillation and Flutter and Congestive Heart Failure under Ischemic Heart Disease -related Mortality, Stratified by Place of Death in Adults in the United States 1999-2023**

| **c** | **Deaths** | | | |
| --- | --- | --- | --- | --- |
| **Year** | **Medical Facility** | **Nursing Home/Long-term Care Facility** | **Home** | **Other/Unknown** |
| **1999** | 2712 | 2503 | 1244 | 162 |
| **2000** | 2736 | 2558 | 1206 | 180 |
| **2001** | 2778 | 2690 | 1334 | 189 |
| **2002** | 2781 | 2562 | 1357 | 245 |
| **2003** | 2822 | 2549 | 1373 | 256 |
| **2004** | 2635 | 2444 | 1508 | 280 |
| **2005** | 2846 | 2831 | 1608 | 281 |
| **2006** | 2794 | 2650 | 1569 | 291 |
| **2007** | 2636 | 2657 | 1647 | 269 |
| **2008** | 2658 | 2640 | 1656 | 290 |
| **2009** | 2412 | 2550 | 1811 | 295 |
| **2010** | 2453 | 2543 | 2000 | 337 |
| **2011** | 2485 | 2664 | 2125 | 336 |
| **2012** | 2474 | 2839 | 2268 | 395 |
| **2013** | 2530 | 2947 | 2549 | 478 |
| **2014** | 2525 | 2994 | 2872 | 413 |
| **2015** | 2643 | 3172 | 2995 | 453 |
| **2016** | 2499 | 2950 | 3199 | 415 |
| **2017** | 2731 | 3094 | 3351 | 477 |
| **2018** | 2806 | 3164 | 3676 | 538 |
| **2019** | 2746 | 3192 | 3794 | 568 |
| **2020** | 2504 | 2727 | 4611 | 615 |
| **2021** | 2745 | 2641 | 4888 | 573 |
| **2022** | 2835 | 2814 | 4527 | 668 |
| **2023** | 2724 | 2924 | 4461 | 550 |
| **Total** | 66510 | 69299 | **63629** | 9554 |
